# Case Report: Pathological Complete Response in a Brain-Metastatic Lung Squamous Cell Carcinoma Patient With Long-Term Benefit From Chemo-Immunotherapy

**DOI:** 10.3389/fonc.2021.693704

**Published:** 2021-09-22

**Authors:** Chen Hu, Qiang Ma, Nengsheng Li, Nuo Luo, Shuai Hao, Minrui Jiang, Fei Pang, Yan Yang, Li Li, Yong He

**Affiliations:** ^1^ Department of Respiratory Disease, Daping Hospital, Army Medical University, Chongqing, China; ^2^ Department of Pathology, Daping Hospital, Army Medical University, Chongqing, China; ^3^ Department of Radiology, Daping Hospital, Army Medical University, Chongqing, China; ^4^ Department of Medical & Products, Origimed, Shanghai, China

**Keywords:** NSCLC, immunotherapy, neoadjuvant therapy, pathological complete response (PCR), immune checkpoint inhibitors (ICI)

## Abstract

Immune checkpoint inhibitors have brought long-term survival benefit in advanced non-small cell lung cancer patients without driver gene mutations. Even after withdrawal of immunotherapy for a maximum of two years, some patients still benefit from this therapy and the reason is not fully clear. Results from several neoadjuvant trials indicated that in resectable lung cancer patients, neoadjuvant immunotherapy or chemo-immunotherapy led to major or complete pathological responses in a high proportion of tumors. Here we report a case of a brain-metastatic lung squamous cell carcinoma patient who received supratentorial tumor resection and thoracic surgery after chemo-immunotherapy, and achieved a pathological complete response (pCR) in both lesions. This case indicated that pCR can also happen in advanced-stage lung cancer patients receiving chemo-immunotherapy, which may be the reason for long-term benefit of those patients.

## Introduction

Immune checkpoint inhibitors (ICIs), represented by anti-programmed cell death-1 (PD-1) and anti-programmed cell death 1igand-1(PD-L1) antibodies, have already become one of the standard treatment options for advanced non-small cell lung cancer (NSCLC) patients without driver gene mutations. The maximum duration of immunotherapy treatment for advanced lung cancer patients is about 2 years (1–3). However, the reasons why some patients still have long-term survival benefits after drug withdrawal is still not completely clear. The result of NADIM trial indicated that resectable lung cancer patients would benefit from neoadjuvant immunotherapy, supported by the high proportion of tumors that achieved a pathological complete response (pCR) (4). Here we report, for the first time, a case of an advanced lung squamous-cell carcinoma patient who received surgical treatment after immunotherapy and achieved a pCR, which may explain the long-term benefit of immunotherapy in advanced-stage patients even after stop of treatment.

## Case Report

**Figure 1:**
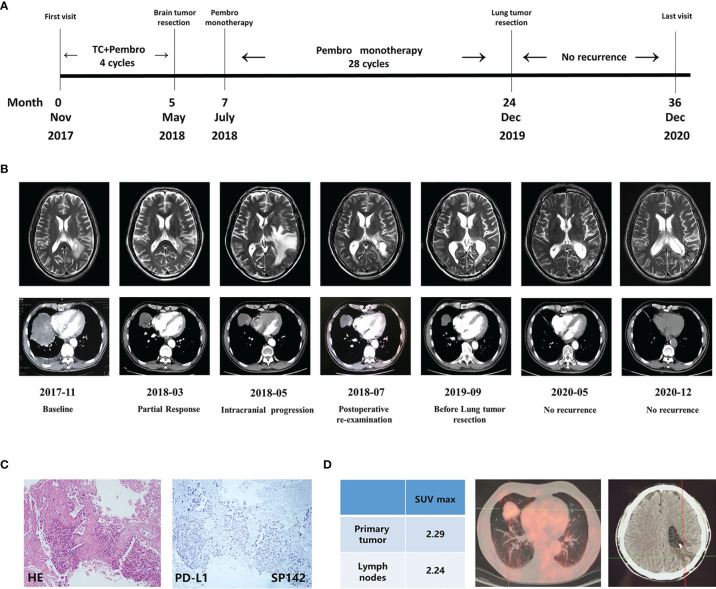
Clinical response to long-term immunotherapy and the effect of surgery. **(A)** Schematics showing the time line of patient’s diagnosis, treatment and response. **(B)** Computed tomography scans and Magnetic resonance imaging showing clinical response to immunotherapy and the effect of surgery. **(C)** The result of PD-L1 staining before immunotherapy. **(D)** PET-CT scan before Lung tumor resection. TC, paclitaxel+carboplatin; Pembro, pembrolizumab.

**Figure 2:**
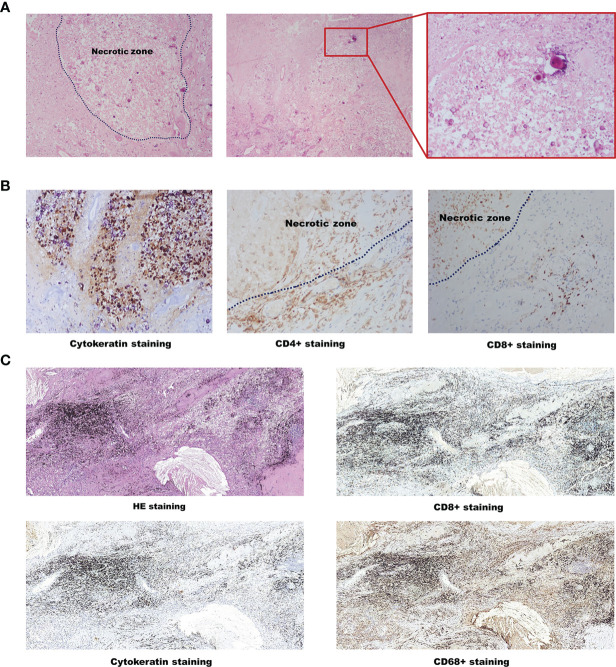
Pathological results of postoperative specimens. **(A)** Hematoxylin-eosin staining of brain tumor tissue. **(B)** CK, CD4^+^ and CD8^+^ staining of brain tumor tissue. **(C)** HE, CD8+, Cytokeratin and CD68+ of lung tumor tissue. HE, Hematoxylin-eosin.

A 61-year-old male patient with a 40 pack-year history of cigarette smoking was admitted to our department in Nov 2017. Chest CT scanning found a right lung mass (about 10cm in diameter) (**Figures 1A, B**) and then the CT-guided percutaneous lung biopsy was performed, revealing a lung squamous cell carcinoma (LSCC). Due to socio-economic factors at that time, the patient could not afford to get PET-CT examination. Abdominal ultrasound and whole-body bone scan showed no evidence of metastatic disease. MRI of the brain showed a space occupying lesion (about 1.5cm*1.8cm) adjacent to the posterior horn of the lateral ventricle, while the patient did not show neurological symptom. The patient was then diagnosed as brain-metastatic right LSCC (T4N1M1c, stage IV). PD-L1 immunohistochemistry was performed using the Roche/Ventana anti-PD-L1 antibody SP142, revealing a negative PD-L1 expression (**Figure 1C**). Molecular analysis using a Next Generation Sequencing (NGS, covering 450 genes with a size of 2.6Mb, Origmed, China) revealed the emergence of TP53 mutation, FGFR3–TACC3 fusion, ATM mutation, KMT2D mutation, EPHB1 mutation, along with a high tumor mutation burden (TMB-H 32.8 muts/Mb). The patient’s general condition was assessed to be satisfactory, evaluated by Eastern Cooperative Oncology Group (ECOG) Performance Status (ECOG PS score=1). Then, a multidisciplinary panel discussion was held, and experts from Neurosurgery Department, Radiotherapy Department, and Thoracic surgery Department were involved. According to NCCN guidelines, stereotactic radiosurgery (SRS) alone is recommended for LSCC patients with limited brain metastases. However, the patient refused to receive SRS since there is no neurological symptom. So, after several rounds of panel meetings, the experts finally reached a consensus on treatment plan to initiate systemic chemo-immunotherapy. The patient then started to receive paclitaxel+carboplatin+pembrolizumab from Dec 2017 and achieved partial response (PR) after receiving four cycles of treatment (**Figures 1A, B**). In May 2018, the patient suffered from a sudden speech disorder and brain MRI examination showed that the intracranial lesion was significantly enlarged, and then he underwent the supratentorial tumor resection in order to alleviate symptoms (**Figures 1A, B**). The pathological diagnosis of brain tumor tissue showed necrotic foci and local tumor necrosis, which tended to be tumor infarction (**Figures 2A, B**). In July 2018, the speech function of the patient was restored well. Chest CT showed that the size of lung lesion was further reduced (**Figure 1B**). Then, another multidisciplinary panel discussion was held, and all experts agree on recommendation of thoracic surgery, since the brain lesion has been resected and the pulmonary tumor was well controlled. However, the patient had just recovered from brain surgery and still suffered from mild headache, so he finally refused to undergo thoracic surgery with the fear of more surgical trauma. Since then, the patient continued to receive pembrolizumab monotherapy for 28 cycles. Until Nov 2019, right lung lesion of the patient remained stable (**Figure 1B**). PET/CT scan indicated that there still existed residual metabolic-active areas within lung lesion without any other organ metastases (**Figure 1D**), while the plasma circulating tumor DNA (ctDNA) testing by (NGS) indicated a negative result. The general condition of the patient was satisfactory (ECOG PS score=1).In order to eradicate lung cancer, the patient finally made the decision to undergo right lung middle lobe resection in Dec 2019. Pathological diagnosis of the lung lesion indicated that no residual tumor cells were found in resected tissue and most of the cells appeared to degenerate and die, with cholesterol crystal deposition, peripheral tissue cell aggregation and lymphocyte infiltration, which achieved a pCR (**Figure 2C**). So the postoperative pathological staging was right LSCC (ypT0N0M0), indicating a favorable outcome of pathologic downstaging by neoadjuvant immunotherapy. Although the patient didn’t receive any treatment after surgery, no reoccurrence of tumor was found during a 1-year follow-up to Dec 2020 (**Figure 1B**).

## Discussion

In this study, we report pCR in a case of a LSCC with brain metastasis, 40 pack-year smoking history, high TMB, and negative PD-L1 expression, after chemo-immunotherapy.

Both TMB and PD-L1 expression are predictive biomarkers for anti-PD-1 therapy. Currently, there are multiple tests for TMB that analyze different genomic regions, with multiple cut-offs for high TMB classification yet no cross-test validation available, making it difficult to stratify patients by tissue TMB. In the current study, TMB of the patient was determined with an NGS covering 450 genes with a size of 2.6Mb (Origmed, China), which was shown to have a satisfactory consistency to detect tissue TMB compared with whole-exome sequencing(WES) tests (5). Besides, it has been reported that cigarette smoke can induce PD-L1 expression on lung epithelial cells (6) and smoker patients have higher expression of PD-L1 than never-smoker patients (7). In a recent study involving 644 NSCLC patients, the percentages of PD-L1 negative patients were 19.0%, 12.8%, and 7.3% in never smoker, former smoker, and current smoker, respectively (8). In the current case, PD-L1 was found negative in both pre-treatment tumor tissue as well as in tumor sections after surgery, which gave us the reassurance that the PD-L1 was negative for this patient. These data indicated that the patient possibly benefited from chemo-immunotherapy due to high TMB.

After 4-cycles of chemo-immunotherapy, the patient developed severe neurological symptoms, so he had to undergo supratentorial tumor resection to relieve the symptoms. In most cases, the aggravation of the neurological symptoms usually indicates the progression of the brain lesions. However in this case, it was surprising that the pathological result of brain tumor surgery showed flaky necrosis areas between the glial background, the necrosis areas were scattered in epithelioid cell remnants and focal calcification, and lymphocytosis was found around the peripheral blood vessels, indicating the brain lesion of the patient achieved pCR just after 4-cycles of chemo-immunotherapy. Considering that the infarction and neurological symptoms were induced by brain edema rather than brain tumor progression, we speculated that immunotherapy was still effective for treating the patient’s brain metastatic lesion, which was in accordance of the previous reports (9). On the other hand, it also reminds us that neurological symptoms may not match the true disease state of patients receiving immunotherapy and the treatment plan will be directly influenced by the evaluation of the actual condition of the brain tumor. So it’s necessary to be more careful when assessing the efficacy of immunotherapy in patients with brain metastases, contributing to distinguish whether the symptoms are caused by tumor progression or necrotic lesions.

The patient recovered well after supratentorial tumor resection and received pembrolizumab monotherapy regularly for 2 years. Lung lesion of the patient kept stable and PET-CT showed no metastases, indicating that he had the opportunity to undergo radical resection of lung cancer. Surprisingly, a pCR response was found on the postoperative pathological examination of lung tumor. Actually the lung lesion of the patient had already obviously diminished (about 10cm in Diameter to 4.6cm*3.6cm) after 4-cycles of chemo-immunotherapy and kept stable without significant changes during the process of pembrolizumab monotherapy. It is also worth wondering whether the pCR of a metastatic lesion could predict pathologic response of primary tumor, which may aid in decision making of the multidisciplinary panel discussion.

Supported by NCCN guidelines, PET-CT is considered to be relatively better method to predict curative effect of neoadjuvant immunotherapy in NSCLC (10, 11). However, the results of preoperative PET-CT, which showed that residual metabolic-active areas existed, were not completely consistent with the result of postoperative pathology, which showed a pCR, indicating that PET-CT maybe not good enough to be an independent predictive marker of pCR in this case. In Neostar study (12), the nodal immune flare has been interpreted as an phenomenon that the CT and PET revealed the nodal progression after immunotherapy, but only noncaseating granulomas found on pathologic examination, indicating that inflammatory effects may influence the metabolic activation and size of tumor. So we speculate that there are numbers of lymphocyte accumulations around the cancer foci accompanied by lymphoid follicle formation, which may be the metabolic-active ingredient suggested by PET-CT. Previous studies have verified the predictive value of ctDNA on the efficacy of PD-1 inhibitor in advanced NSCLC, suggesting that ctDNA has the potential to be a predictor of treatment response (13, 14). It is worth noting that the preoperative plasma test indicated a negative result in this case. ctDNA shouldn’t be considered as an significant predictive indicator of pathological response, while it can reflect tumor burden and guide treatment options as a good complement to PET-CT. Therefore, the prediction of histopathologic response in patients with NSCLC after neoadjuvant immunotherapy may be more accurate when defined by using both ctDNA and FDG-PET together rather than separately.

Finally, it’s also worth considering that even though the patient didn’t receive any treatment after radical resection of pulmonary carcinoma and stopped immunotherapy for nearly 1.5 years in the current case, no reoccurrence of tumor was found in the latest re-examination and he has survived for more than 3.5 years since the first detection of advanced LSCC. The result of CA-209003, which has the longest follow up reported so far, indicated that 5-year OS rate was 16% for ICIs-treated NSCLC patients, while it is unknown why some patients still have long-term survival benefits after stopping after chemo-immunotherapy in this clinical trial (15). Through the analysis of this case, we speculate that achieving pCR might be the reason for the long-term benefit of the patients after stopping immunotherapy.

In summary, we present the first case of an advanced LSCC patient with brain metastasis who achieved a pCR after chemo-immunotherapy, which may guide treatment options and explain the reasons why some patients still have long-term survival benefits even after chemo-immunotherapy withdrawal.

## Data Availability Statement

The original contributions presented in the study are included in the article/supplementary material. Further inquiries can be directed to the corresponding authors.

## Ethics Statement

The studies involving human participants were reviewed and approved by the ethics committee of Daping Hospital, Army Medical University. Written informed consent was obtained from the participant for the publication of this case report.

## Author Contributions

YH, LL, and CH worked on the conception and design of the case report. CH, LL, NLi, NLu, and SH collected data on patient- reported outcome. QM performed analysis of pathologic results. MJ collected image data of the patient. FP and YY performed molecular analysis. YH, CH, LL and QM wrote and revised the manuscript. All authors contributed to the article and approved the submitted version.

## Funding

This work was supported by a National Natural Science Foundation of China (81972189, 81672287). Funding from Daping Hospital (2019CXLCA003, 2019CXLCB011).

## Conflict of Interest

Authors FP and YY were employed by Origimed.

The remaining authors declare that the research was conducted in the absence of any commercial or financial relationships that could be construed as a potential conflict of interest.

## Publisher’s Note

All claims expressed in this article are solely those of the authors and do not necessarily represent those of their affiliated organizations, or those of the publisher, the editors and the reviewers. Any product that may be evaluated in this article, or claim that may be made by its manufacturer, is not guaranteed or endorsed by the publisher.
